# Epitaxial Growth of Y_2_O_3_ Buffer Films on Biaxially Textured Ni-W Tapes via a Photochemical Method

**DOI:** 10.3390/ma19214593

**Published:** 2026-10-30

**Authors:** Jianshe Yue, Zhen Li, Huimin Cai

**Affiliations:** 1 School of Chemistry and Chemical Engineering, Xianyang Normal University, Xianyang 712000, China; yuejs0204@163.com (J.Y.); lizhen0408@163.com (Z.L.); 2 School of Industry and Information Technology, Hainan College of Vocation and Technique, Haikou 570216, China

**Keywords:** sol–gel, UV irradiation, photochemical, buffer film, YBCO superconductor, Y_2_O_3_ film, Ni-W tape

## Abstract

**Highlights:**

- UV photochemical treatment removes residual carbon and suppresses defects in sol–gel Y_2_O_3_ films.
- UV-pretreated Y_2_O_3_ buffer films show fine grains, low roughness, and strong biaxial texture.
- Single-layer Y_2_O_3_ buffer replicates the Ni-W texture and enables epitaxial YBCO growth.
- YBCO films on the photochemical Y_2_O_3_ buffer reach a self-field Jc of 2.65 MA/cm^2^ at 77 K.
- A vacuum-free photochemical sol–gel route lowers the cost of YBCO-coated conductors.

**What are the main findings?**

- Photochemical treatment decomposes organic groups and removes residual carbon in sol–gel Y_2_O_3_ precursor films, suppressing volatilization-induced defects.
- The UV-pretreated Y_2_O_3_ buffer film is dense and crack-free with fine grains and good in-plane biaxial texture (FWHM 1.6–1.9°).
- Epitaxial YBCO grown on this single-layer buffer exhibits a self-field Jc of 2.65 MA/cm^2^ at 77 K with typical flux-pinning behavior.

**What are the implications of the main findings?**

- The findings reveal that a vacuum-free, photochemical-assisted sol–gel route is capable of fabricating high-quality single-layer Y_2_O_3_ buffer films on biaxially textured Ni–W tapes, which avoids complicated multi-stacked buffer strucutres and vacuum deposition techniques.
- This strategy simplifies the manufacturing workflow and cuts the production cost of YBCO-coated conductors, which is beneficial for their large-scale and low-cost practical applications.
- The underlying photochemical mechanism, namely low-temperature decomposition of organics species and efficient elimination of residual carbon, can be extended to other oxide buffers layers and solution-processed functional thin-film systems.

**Abstract:**

Buffer layers are indispensable components for YBa_2_Cu_3_O_7−δ_(YBCO)-coated conductors deposited on biaxially textured Ni-W metallic substrates, which act as diffusion barrier and epitaxial growth templates for superconducting films. In this work, both the Y_2_O_3_ single buffer film and the subsequent YBCO superconductor film were successfully fabricated on cube-textured Ni-W tapes via a facile photochemical-assisted sol–gel method. Specifically, the precursor gel films of Y_2_O_3_ were pretreated by ultraviolet (UV) irradiation. High-energy UV photons break organic chemical bonds and generate ozone and active oxygen radicals, which effectively decompose organic components and eliminate residual carbon at a low temperature. This photochemical treatment suppresses pore defects caused by the violent volatilization of organic substances during high-temperature annealing. The UV-treated Y_2_O_3_ buffer films present fine uniform grains and a low surface roughness of 5.46 nm, possessing excellent biaxial texture. The single-layer Y_2_O_3_ buffer can perfectly inherit the texture characteristics of the Ni-W substrate and provide a high-quality epitaxial growth template. On this sol–gel-derived Y_2_O_3_ buffer layer, the sol–gel-prepared YBCO superconducting film achieves high-quality in-plane epitaxial growth. The optimized YBCO-coated conductor exhibits a self-field critical current density of 2.65 MA/cm^2^ at 77 K with a superconducting transition width of 2.0 K, which confirms excellent superconducting performance. This vacuum-free photochemical-assisted sol–gel strategy provides a low-cost and promising technical route for the fabrication of high-quality single-buffer-layer YBCO-coated conductors.

## 1. Introduction

The development of second-generation higher-temperature superconductors (HTSs) has attracted considerable attention owing to their high critical temperature, high current density, and compact dimensions, which endow them with great potential for power-engineering, medical and nuclear-physics applications [1,2,3]. Among these HTS materials, YBa_2_Cu_3_O_7−δ_ (YBCO) is a promising candidate for power-engineering applications [4,5,6]. Over the past few years, extensive research efforts have been devoted to the fabrication of YBCO film conductors by different methods [7,8,9]. Among these preparation methods, the sol–gel method is simple, reliable, and feasible for engineering-scale implementation. The fabrication of engineering-scale superconducting tapes requires rolling-assisted biaxially textured (RABiTS) Ni-W metallic substrates, onto which superconducting materials are deposited. However, YBCO cannot be directly deposited on Ni-W substrates for two reasons. First, the sol–gel process involves subsequent high-temperature heat treatment. During this process, metallic elements readily diffuse into the superconducting film and degrade its superconducting performance. Second, grains in Ni-W tapes possess a well-defined crystallographic texture. Serving as a template, the substrate induces epitaxial growth of YBCO with grain misorientation angles below 10°, so as to achieve optimal electrical-conductivity performance.

Therefore, an intermediate buffer film is required between the superconducting film and the Ni-W substrate. This buffer film serves two functions: first, it acts as a diffusion barrier film; second, it functions as a template to induce epitaxial growth of the superconducting film on its surface, thereby assuring that the grain-boundary misorientation of YBCO remains below about 10°. The buffer films must possess lattice parameters well-matched with both the metal Ni-W substrate and YBCO. Over the past decade, numerous suitable materials including CeO_2_, Y_2_O_3_, BaZrO_3_, yttria-stabilized zirconia (YSZ) and La_2_Zr_2_O_7_ have been reported for buffer-film applications [10,11,12,13]. In these studies, the typical design adopts multi-stacked oxide buffer films, such as CeO_2_/YSZ/CeO_2_ or CeO_2_/YSZ/Y_2_O_3_ [14,15]. Nevertheless, fabricating such multi-stacked buffer structures increases manufacturing costs and complicates the preparation procedure. Moreover, the epitaxial quality of the buffer films can deteriorate with increasing thickness, because buffer materials exhibit lattice-constant mismatches relative to the metallic substrate [16]. Accordingly, it is desirable to develop a single-film buffer film. Y_2_O_3_, as a promising buffer-film candidate, has drawn considerable attention [17]. It adopts a bixbyite crystal structure with a lattice parameter of 10.607 Å and Y-Y atomic spacing of 3.75 Å. The small lattice mismatch enables epitaxial growth of YBCO on the Y_2_O_3_ buffer film. Meanwhile, Y_2_O_3_ exhibits favorable chemical compatibility with the Ni-W substrate. To date, Y_2_O_3_ buffer films have been deposited on Ni-W tapes by various vacuum-based techniques [18,19]. However, the high cost and complexity of vacuum-deposition technologies restrict the fabrication of long-length HTS tapes. Sol–gel technique has been adopted for buffer-film preparation, as it is a low-cost, straightforward approach requiring no vacuum equipment. Furthermore, sol–gel method allows precise compositional control of final products and the manufacture of long-length tapes. Hence, sol–gel method is considered one of the promising routes for producing low-cost coated conductors. Generally, sol–gel method requires high-temperature treatment to decompose precursor gel films. During thermal processing, volatilization of organic species from the gel film generates porous defects, which impair the epitaxial growth of buffer films. To obtain dense buffer films and reduce the heat treatment temperature, this work employs a photochemical approach combined with UV irradiation to prepare Y_2_O_3_ buffer films. Photochemical treatment of sol–gel precursors can effectively decompose organic functional groups, lower the crystallization temperature of films, and suppress inter-diffusion of metallic elements into buffer films [20,21,22]. The as-prepared Y_2_O_3_ buffer films exhibit epitaxial grains, dense microstructure, uniform grain size and fine grains. More importantly, the photochemical method can substantially lower the grain-growth temperature of Y_2_O_3_ [23,24,25], thereby preventing metal-element diffusion into the buffer film. YBCO superconducting films grow with preferred orientation on the buffer layer of single-sided Ni-W tapes. The resulting YBCO/Y_2_O_3_/Ni-W architecture demonstrates favorable superconducting performance.

## 2. Materials and Methods

The Y_2_O_3_ precursor solution was prepared using yttrium acetate as the raw material, and a mixture of methanol and lactic acid was adopted as the solvent. The molar ratio of methanol to lactic acid was 5:1. Lactic acid acts as a chelating agent to improve the stability of the sol and inhibit the rapid hydrolysis of the precursor. Yttrium acetate was dissolved in the mixed solvent and stirred at 70 °C for 2 h to obtain a transparent homogeneous solution, after the sol is formed, it is aged at room temperature for 24 h to achieve sufficient homogenization. The concentration of Y^3+^ ion was fixed at 1 mol/L. The Ni-W alloy tapes (Evico GmbH, Dresden, Germany) were used as the substrate. Before sol coating, the tapes were ultrasonically cleaned with absolute ethanol to remove surface organic contaminants and subsequently heat-treated at 900 °C for 1 h in a mixed N_2_ and H_2_ atmosphere (N_2_ 95 vol%, H_2_ 5 vol%) to eliminate surface oxide films. Y_2_O_3_ gel films were deposited via the dip-coating method with a withdrawal speed of 3 mm/s. To make the experiment closer to industrial production, the gel film was coated only on one side of the Ni-W substrate, while the other side was wiped off. The as-coated Y_2_O_3_ precursor films were first dried at 50 °C for 30 min to evaporate the organic solvent, this temperatures can suppress evaporation induced by vigorous boiling, which otherwise causes bubbles, pinholes and cracks in the gel film, and then photochemically treated under ultraviolet (UV) light (Model L2002A3, Ossila Ltd., Sheffield, UK. 185 nm: 10%, 254 nm: 90%) for 1 h. To increase the film thickness, the dip-coating, drying and UV irradiation procedures were repeated three times. Finally, the multilayer Y_2_O_3_ gel films were annealed at 700, 800 and 900 °C for 2 h in a humidified N_2_-H_2_ mixed atmosphere (N_2_ 95 vol%, H_2_ 5 vol%) with a heating rate of 10 °C/min.

The YBCO precursor solution was prepared using yttrium acetate, barium acetate, copper acetate as raw materials, with methanol serving as the primary solvent [26,27]. Yttrium acetate was dissolved in a mixed solution of methanol and diethylenetriamine at a mole ratio 1:1. Barium acetate was dissolved in a methanol–lactic acid mixture, where the mole ratio of Ba^2+^ to lactic acid was 1:2. Copper acetate was dissolved in methanol and acrylic acid with a Cu-to-acrylic acid mole ratio of 1:3. The three individual solutions were thoroughly mixed to form the homogeneous YBCO precursor solution, with a total metal ion concentration of 2 mol/L. YBCO gel films were deposited on the Y_2_O_3_-buffered Ni-W tapes by dip-coating at a withdrawal speed of 2 mm/s. The obtained gel films were pyrolyzed at 450 °C for 1 h in humidified oxygen, and subsequently annealed at 780 °C for 2 h in a humidified O_2_-N_2_ mixed atmosphere (O_2_ 80 vol%, N_2_ 20 vol%), the final YBCO film obtained has dimensions of 20 mm in length, 10 mm in width and 0.68 μm in thickness.

The phase composition and crystallographic orientation of Y_2_O_3_ buffer film were characterized by X-ray diffraction (XRD: Bruker AXS SE, Karlsruhe, Germany). The in-plane texture of Y_2_O_3_ and YBCO film was characterized by XRD Phi scan (Rigaku Corporation, Akishima, Japan). The surface morphologies of Y_2_O_3_ buffer film and YBCO superconducting film were observed using a field-emission scanning electron microscope (FE-SEM, JEOL Ltd., Akishima, Japan, JEOL-6700F). The surface roughness of the Y_2_O_3_ film was characterized by atomic force microscopy (AFM: Bruker Corporation, Billerica, MA, USA). The magnetic properties of the YBCO films were measured by a multi-function vibrating sample magnetometer (VersaLab-VSM: Quantum Design, Inc, San Diego, CA, USA). Fourier transform infrared (FT-IR) spectroscopy (Bruker AXS SE, Karlsruhe, Germany, VERTEX70) was employed to characterize functional group structures of precursor films. The four-probe (Quantum Design, Inc., San Diego, CA, USA) method was adopted to characterize the Jc and resistance–temperature (R-T) curves of YBCO-coated conductors.

## 3. Results

### 3.1. XRD Analysis

**Figure 1:**
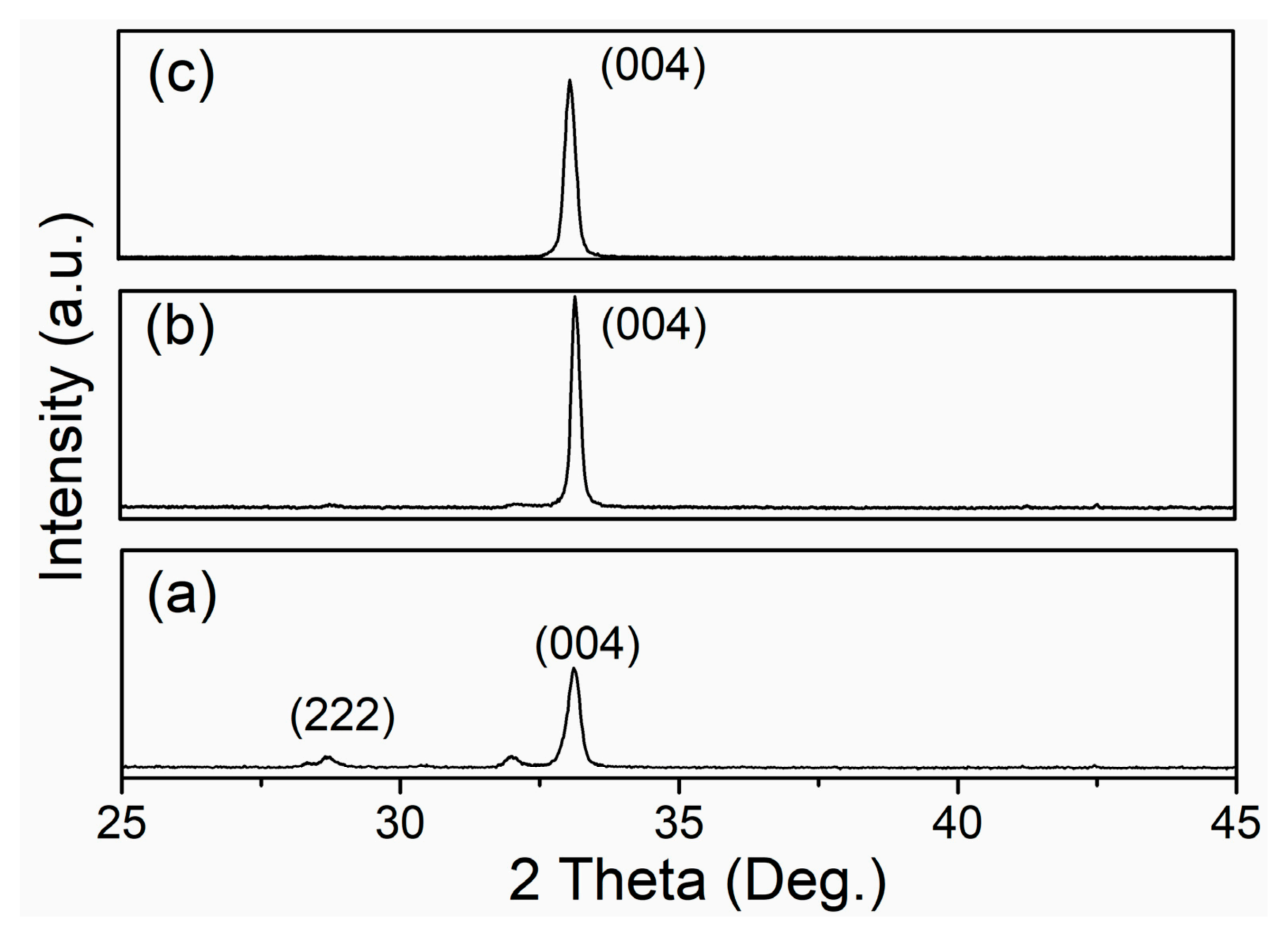
XRD of Y_2_O_3_ films annealed at different temperatures: (**a**) 700 °C, (**b**) 800 °C, and (**c**) 900 °C.

The Y_2_O_3_ precursor film was deposited on a cube-textured Ni-W metal substrate. After drying at 50 °C, the film was subjected to UV irradiation (wavelength of 185 nm (10%) and 254 nm (90%)) for 1 h. It was subsequently heat-treated at 700 °C, 800 °C and 900 °C for 2 h, respectively. The XRD patterns of the resulting films are shown in Figure 1. After heat treatment at 700 °C, the desired dominant (400) peak emerged, accompanied by the impurity (222) peak. When the annealing temperature was increased to 800 °C, a remarkable reduction in the intensity of the impurity (222) peak is observed, and the main (400) peak remained. After annealing at 900 °C, the film exhibited a single (400) diffraction peak. However, the full width at half-maximum (FWHM) of the Y_2_O_3_ film annealed at 900 °C was larger than that of the sample treated at 800 °C. This suggests that relatively coarse Y_2_O_3_ grains were formed upon annealing at 800 °C. According to the Arrhenius law [28], the atomic diffusion coefficient increases exponentially with temperature. Therefore, Y and O atoms have higher mobility at 900 °C, which normally promotes grain growth. Nevertheless, the cube-textured Ni-W substrate acts as an epitaxial template and provides abundant nucleation sites at elevated temperatures. The enhanced nucleation rate outweighs the grain-growth process. Consequently, fine grains with strong (400) preferred orientation are obtained at 900 °C.

### 3.2. Microstructural Analysis

**Figure 2:**
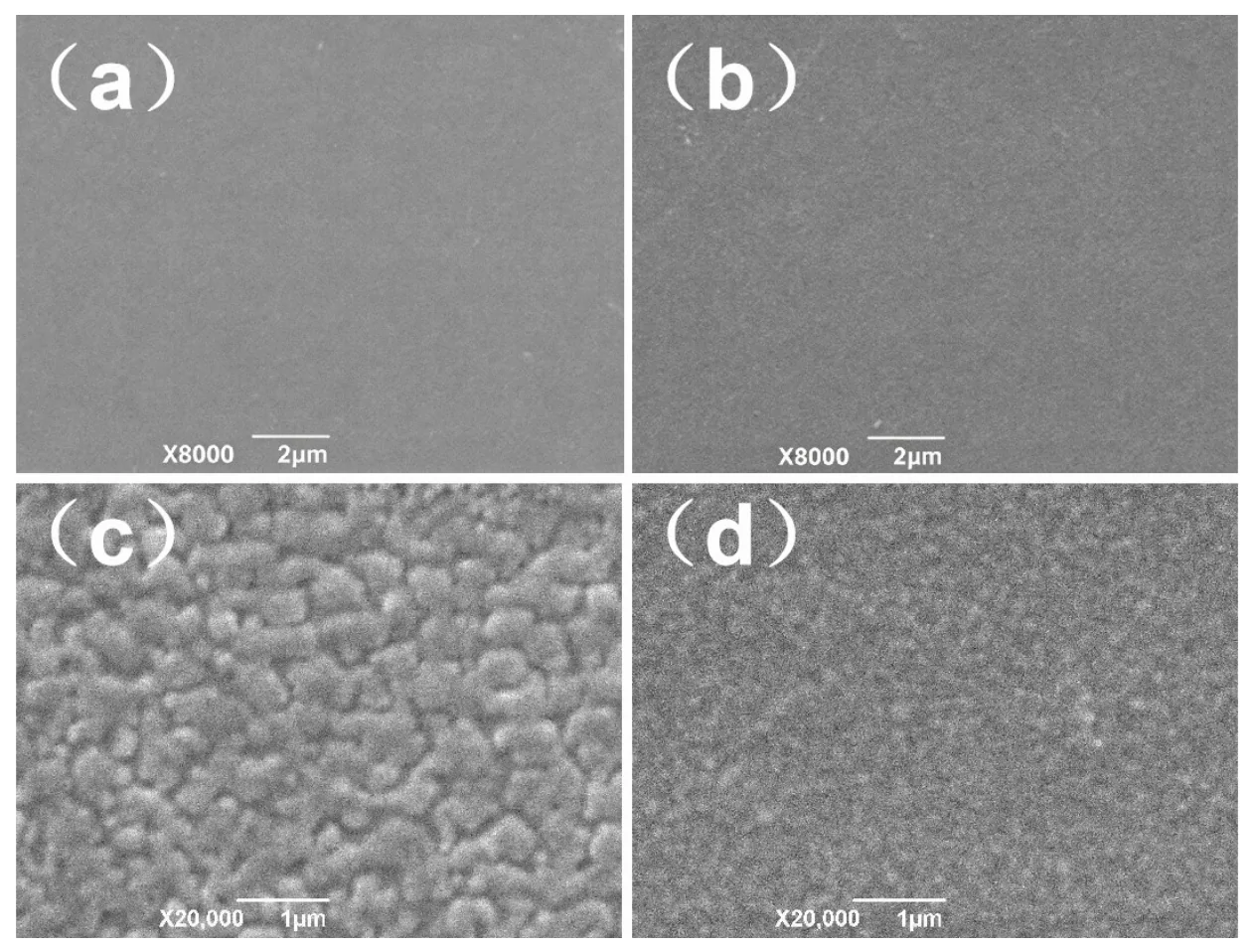
SEM images of Y_2_O_3_ buffer film annealed at 900 °C: (**a**) low-magnification of no-UV-irradiated, (**b**) low-magnification of UV-irradiated, (**c**) high-magnification of no-UV-irradiated (**d**) high-magnification of UV-irradiated.

**Figure 3:**
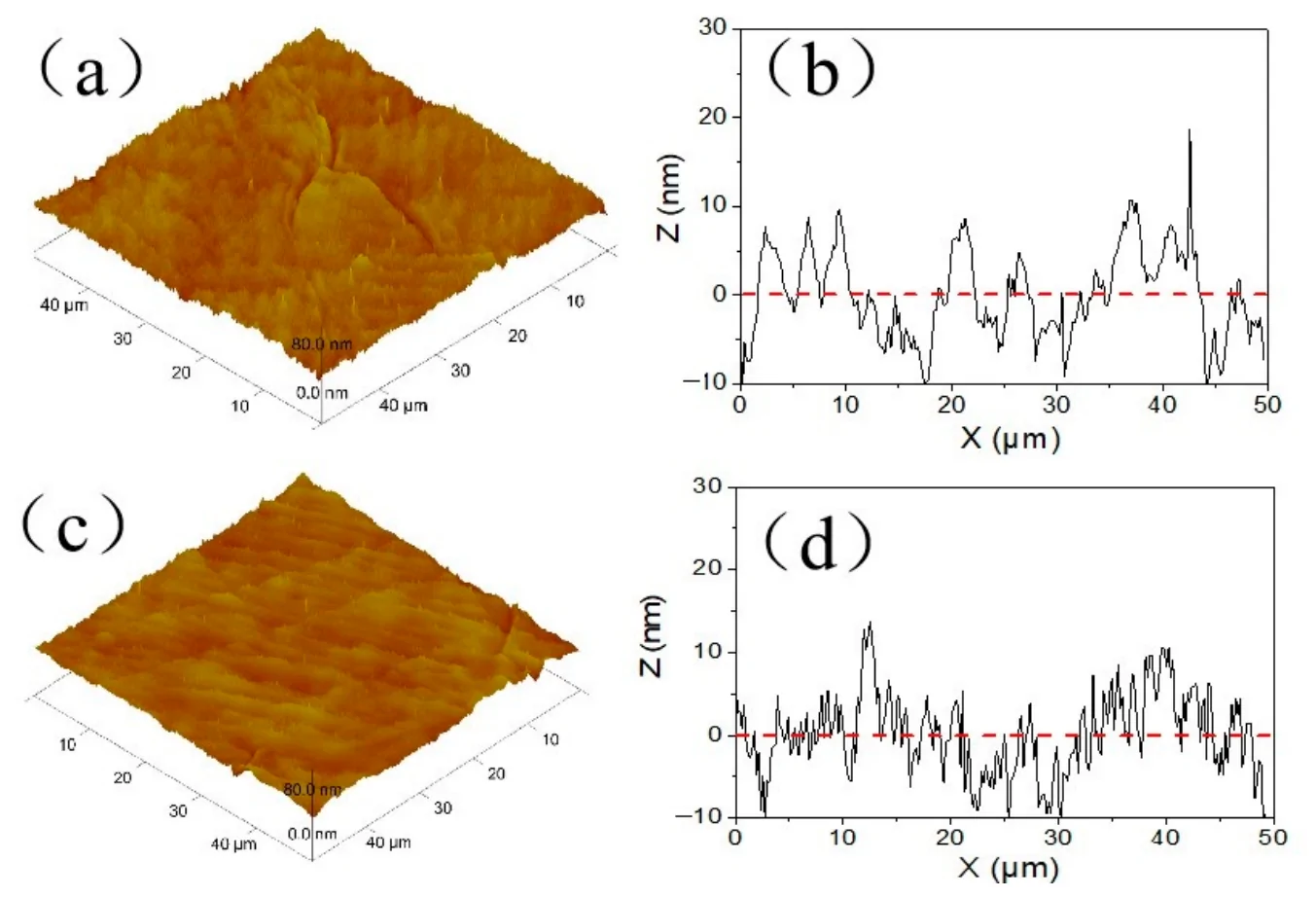
AFM of Y_2_O_3_ buffer film annealed at 900 °C: (**a**) no-UV-irradiated 40 μm × 40 μm 3D surface topography; (**b**) no-UV-irradiated 2D roughness distribution map, (**c**) UV-irradiated 40 μm × 40 μm 3D surface topography, (**d**) UV-irradiated 2D roughness distribution map.

The microstructure of the Y_2_O_3_ buffer film annealed at 900 °C was characterized by SEM, as shown in Figure 2. To highlight the effect of UV irradiation on the films, comparative observation was carried out on the Y_2_O_3_ buffer film annealed at the same temperature without UV pretreatment. Figure 2a,c present the micrographs of the non-UV-irradiated sample, while Figure 2b,d correspond to the sample subjected to prior UV irradiation. In the low-magnification view (Figure 2a,b), both samples exhibit flat surfaces, and no obvious defects can be observed. However, under high-magnification observation (Figure 2c,d), significant differences can be observed. The no-UV-irradiated Y_2_O_3_ buffer film displays clearly coarse grains with large-angle grain boundaries. By contrast, the UV-irradiated Y_2_O_3_ buffer film consists of fine, uniform grains with predominantly small-angle grain boundaries. The surface roughness of the two types of Y_2_O_3_ films prepared by different processes was characterized by AFM, and the results are shown in Figure 3. The 3D topography images obtained from 40 μm × 40 μm scanning of the Y_2_O_3_ samples are shown in Figure 3a,c. It can be clearly observed that the non-UV-irradiated Y_2_O_3_ sample possesses coarse grains with prominent surface undulations. In contrast, the UV-irradiated Y_2_O_3_ sample exhibits a much smoother surface with reduced topographic fluctuations. The roughness distributions derived from 2D scanning of the two samples are presented in Figure 3b,d, and the summarized roughness parameters are listed in Table 1. The maximum surface roughness of the non-UV-irradiated Y_2_O_3_ sample is 19.8 nm, whereas the value for the UV-irradiated counterpart is only 5.46 nm.

### 3.3. FT-IR Analysis of Gel Film

**Figure 4:**
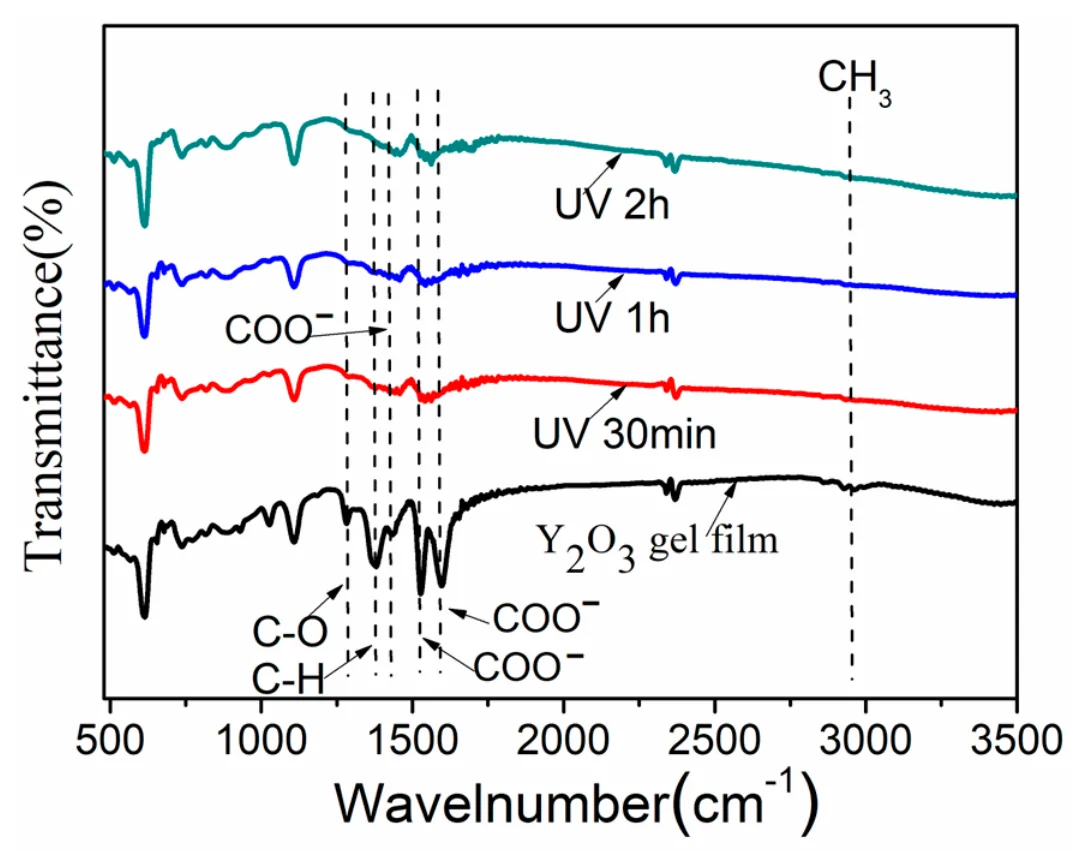
FT-IR transmission spectra of Y_2_O_3_ gel film. Symmetric stretching vibration COO^−^ of carboxylate groups from coordinated lactate 1384 cm^−1^.

Evidently, UV irradiation is beneficial to the oriented growth of Y_2_O_3_ grains, the formation of low-angle grain boundaries, and the improvement in surface flatness. To further reveal the underlying mechanism, infrared spectroscopic analysis was performed on Y_2_O_3_ thin films subjected to different UV-irradiation durations, and the results are shown in Figure 4. The CH_3_ peak corresponds to the C-H stretching vibration of methyl groups in acetate and lactic acid. The COO^−^ band at 1580 cm^−1^ is attributed to the asymmetric stretching vibration of carboxylate. The band at 1432 cm^−1^ is assigned to the symmetric stretching vibration of carboxylate. The COO^−^ peak at 1528cm^−1^ originates from the asymmetric stretching vibration of lactate ligands bidentate-chelated with Y ions. The C-H band represents the C-H stretching vibration of methyl groups in lactate. In addition, although C-C bonds are undoubtedly present, the stretching vibration of C-C single bonds appears as weak bands in the fingerprint region. This signal overlaps with the C-O stretching vibration. Moreover, the dipole moment change in C-C bonds is minimal, leading to weak absorption intensity. The peak intensity gradually decreases with increasing UV-irradiation time. After 30 min of UV irradiation, the aforementioned absorption bands nearly disappeared. Further prolonging the irradiation time causes no obvious changes in other spectral bands. These results demonstrate that adsorbed water and carbonate species can be effectively removed after 30 min of UV treatment, accompanied by the decomposition of yttrium acetate.

**Figure 5:**
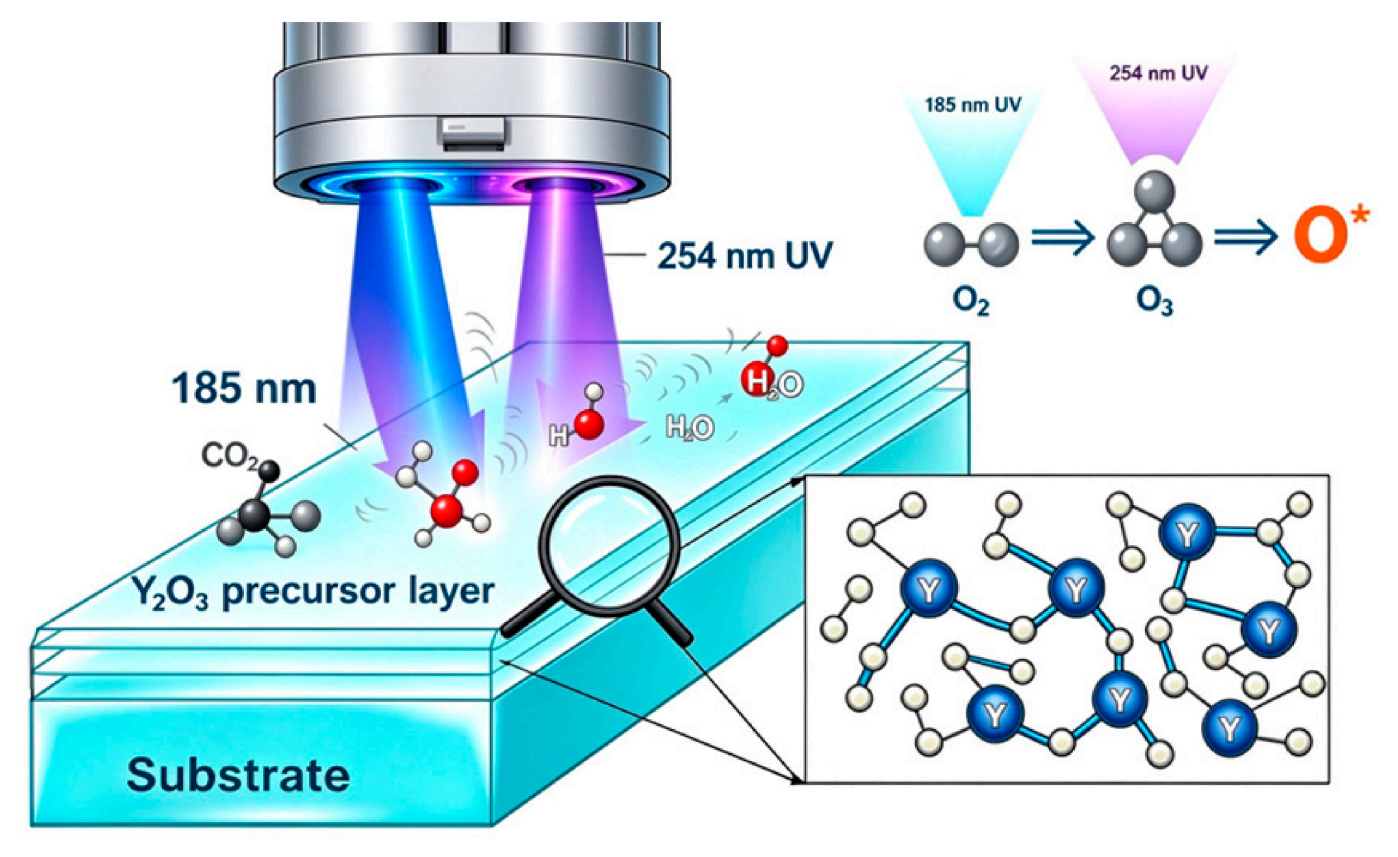
Schematic diagram of UV-irradiated Y_2_O_3_ precursor film.

The UV wavelengths adopted in this work are 185 nm and 254 nm, with corresponding photon energies of 647 kJ/mol (185 nm) and 472 kJ/mol (254 nm), respectively. Both energy values are higher than the bond energies of C-H (413 kJ/mol), H-O (463 kJ/mol), C-O (352 kJ/mol) and C-C(348 kJ/mol) [23]. During irradiation, photochemical reaction occurs, high-energy UV light cleaves organic chemical bonds to form highly active O-M-O bonds. Owing to the bond-cleavage effect of UV irradiation, the organic species inside the film are fully decomposed at a low temperature, which prevents rapid volatilization of solvent and adsorbed water. As a result, fewer defects are formed in the film layer. Furthermore, owing to the effective penetration of UV light and its uniform decomposition of organics, agglomeration of decomposition products is inhibited, ensuring fine and uniform Y_2_O_3_ grains (as shown in Figure 2d). If the Y_2_O_3_ gel film is directly thermally decomposed without prior UV irradiation, water volatilization during heating will induce defects in the film. The escape of carbon species after organic carbonization can also give rise to film defects. Moreover, inhomogeneous heating results in decomposition products of diverse sizes, which are prone to agglomeration. Consequently, coarse Y_2_O_3_ grains are formed, accompanied by large surface undulations of the film (as shown in Figure 2c). More importantly, highly oxidative O_3_ and active oxygen radicals O* generated via photochemical reactions (as shown in Equations (1)–(3)) oxidize and decompose organic components. Gaseous products such as CO_2_ and H_2_O are produced and slowly and gently released from the film, leading to the formation of flat and dense Y_2_O_3_ film. The corresponding schematic diagram is shown in Figure 5.

Equation for O_3_ and O* formation under UV irradiation in air:

O_2_ + hv (185 nm) → O + O, (1)

O_2_ + O → O_3_, (2)

O_3_ + hv (254 nm) → O* + O_2_, (3)

**Figure 6:**
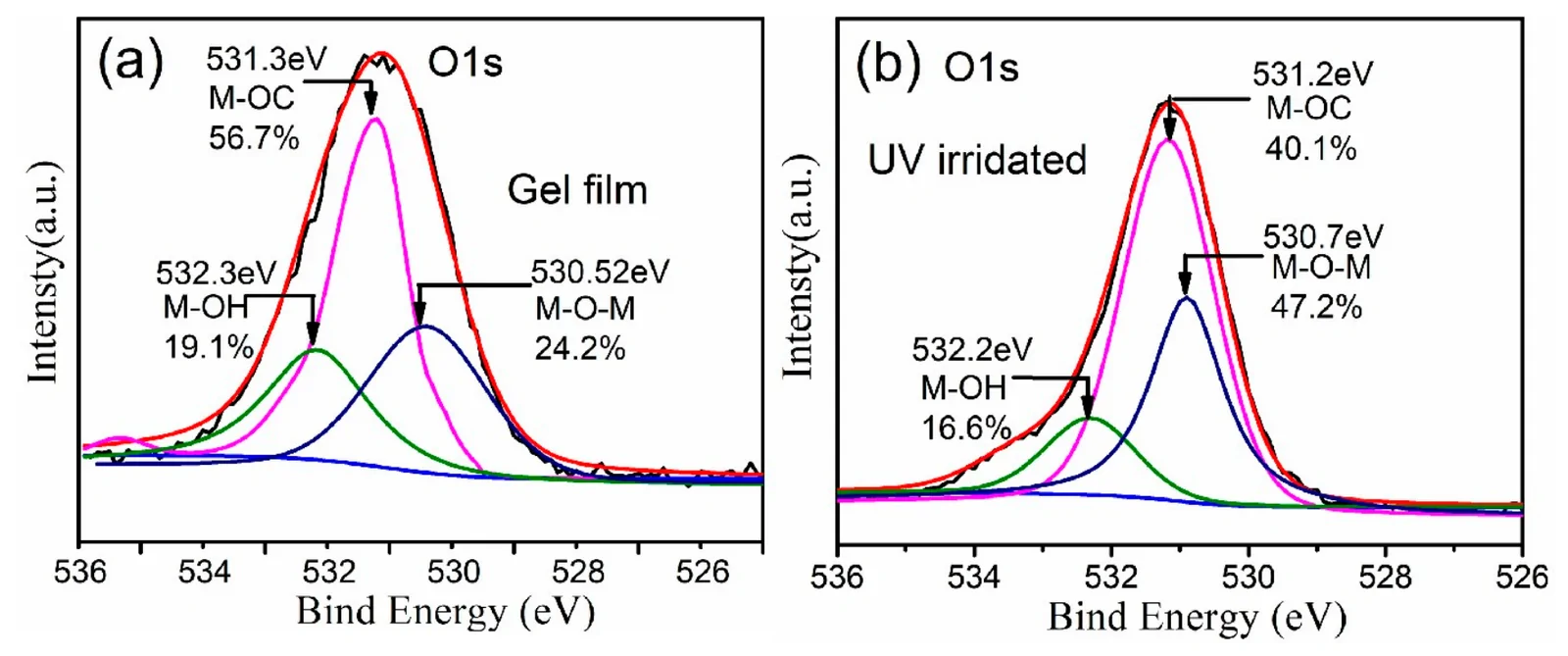
XPS of Y_2_O_3_ gel film: (**a**) no-UV-irradiated; (**b**) UV-irradiated.

Through the above-mentioned photochemical reactions, UV light can effectively promote the decomposition of organics in the Y_2_O_3_ gel film. In addition, by-produced O_3_ and active oxygen radicals O* can also effectively remove the residual carbon in the form of CO_2_. To verify the promoting effect of UV irradiation on organic decomposition in Y_2_O_3_ gel films, XPS analysis was performed on the films before and after UV irradiation. XPS spectra were deconvoluted using CasaXPS software. The O 1s spectra of Y_2_O_3_ were recorded as shown in Figure 6. A clear decrease in Y-OH (M-OH) and Y-OC (M-OC) contents can be seen in the spectra (Figure 6b), accompanied by an increase in Y-O bond content. The increase in the integrated area fraction of the M-O-M component after UV irradiation demonstrates the rise in the relative content of Y-O-Y species on the near-surface region of the film. UV irradiation triggers photochemical decomposition of organic ligands and removes Y-OC organic oxygen species. Consequently, yttrium ions combine with oxygen to form Y-O-Y metal–oxygen bonds. It should be noted that integrated peak area fractions were adopted for quantitative analysis. This demonstrates that UV irradiation can effectively promote the decomposition of the organics in the film layer and remove residual carbon within the film. This reduces defects induced by the oxidative volatilization of carbon and guarantees the integrity of the Y_2_O_3_ film.

**Figure 7:**
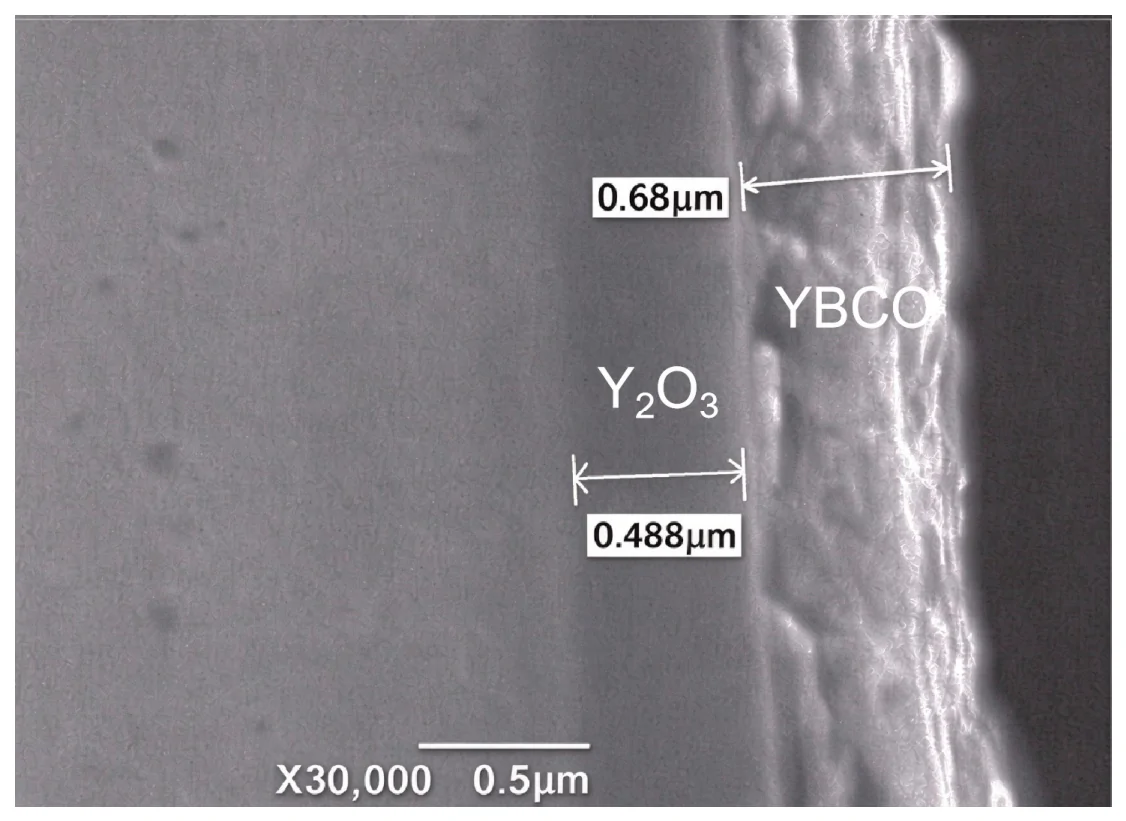
Cross-sectional SEM image of the Y_2_O_3_ buffer film and YBCO film.

After the UV-irradiated Y_2_O_3_ gel film underwent high-temperature heat treatment at 900 °C, Y-O bonds undergo crystallization and yielded crystalline Y_2_O_3_ thin films. To facilitate the overall observation, we deposited a YBCO superconducting layer on the Y_2_O_3_ film and characterized the cross-section of the whole stack by SEM, as shown in Figure 7. The Y_2_O_3_ thin film exhibits a uniform thickness and dense microstructure, and no defects such as pores or cracks are observed. In this work, a continuous and dense Y_2_O_3_ buffer layer with a thickness of approximately 0.488 μm can be obtained after three coating cycles. This thickness is sufficient to block the diffusion paths of metallic elements. However, single- or double-layered Y_2_O_3_ films may contain tiny pinholes or hardly observable defects such as microcracks, which cannot effectively hinder the outward diffusion of Ni and W from the Ni-W substrate. Further increasing the number of coating cycles will introduce larger residual stress in the thicker Y_2_O_3_ film and consequently lead to film cracking. Meanwhile, more coating cycles will increase the fabrication cost. Therefore, three coating cycles represent the optimized processing condition in this study.

### 3.4. The Microstructure, Crystal Structure and Electrical Property of YBCO Film

**Figure 8:**
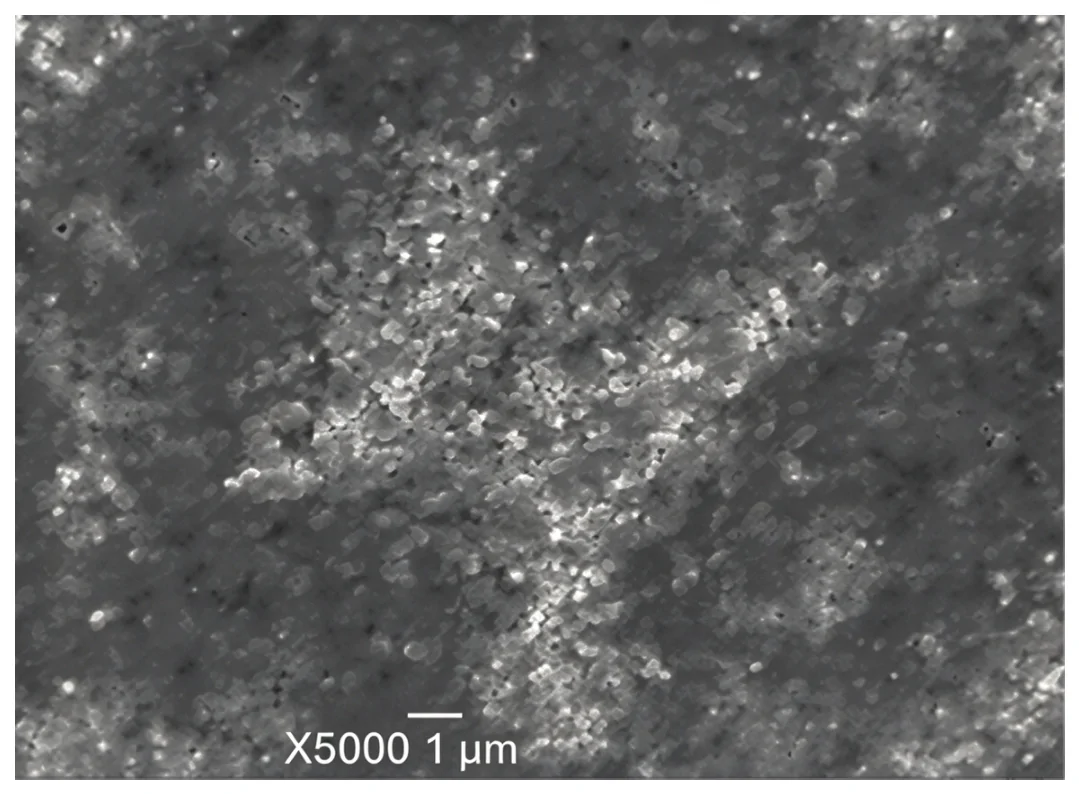
The SEM image of YBCO conducting film.

After the Y_2_O_3_ buffer film was prepared, the YBCO superconducting film was deposited on the surface of the Y_2_O_3_ buffer film. The SEM morphology of the YBCO is shown in Figure 8, and the cross-sectional SEM image is shown in Figure 7. The surface of YBCO film exhibits a flat, dense surface, and no defects such as pores are observed. The cross-sectional view reveals a dense YBCO film layer without observable defects such as pores and microcracks; the average thickness is approximately 0.68 μm.

**Figure 9:**
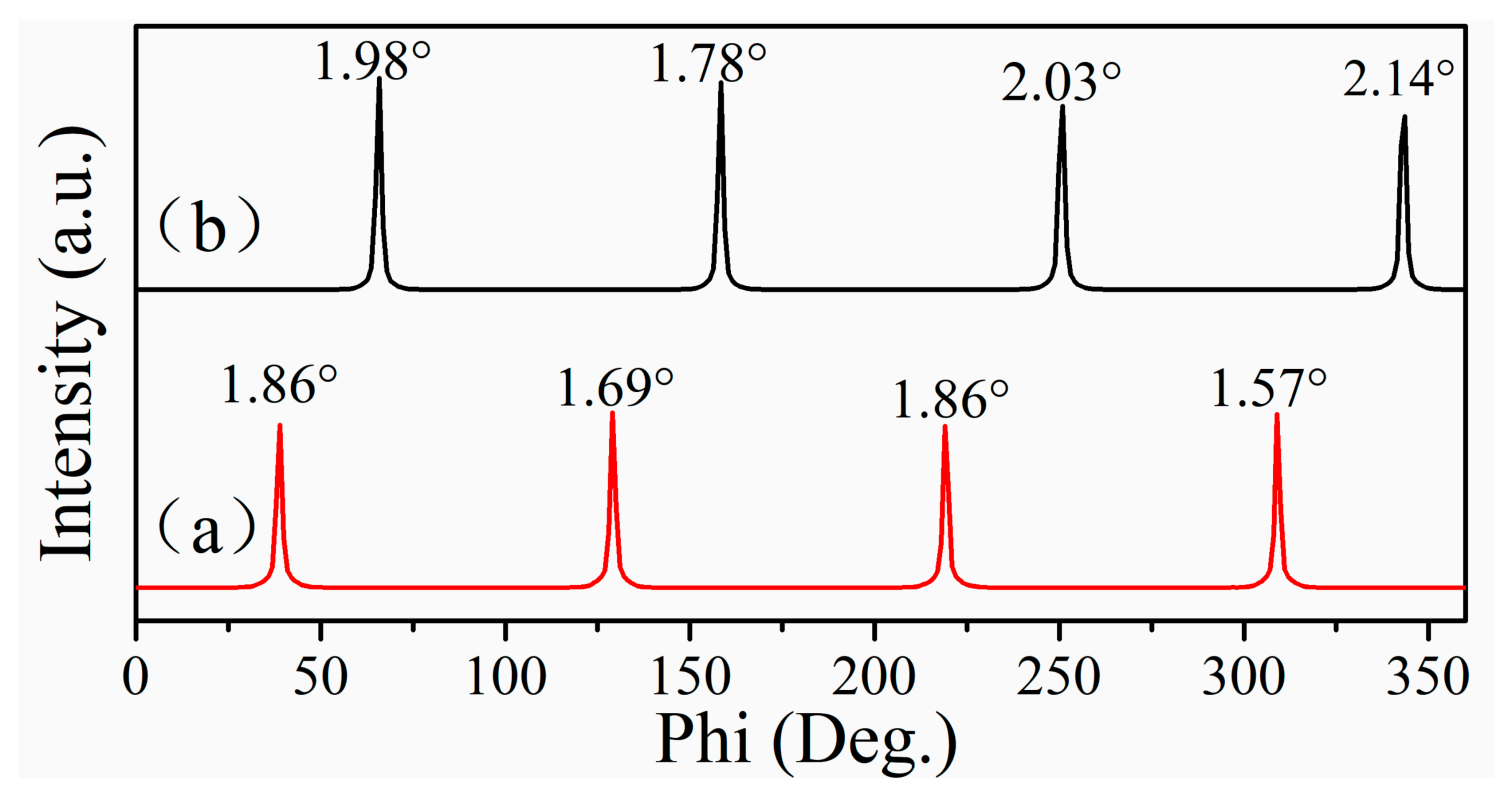
Phi scan of (**a**) Y_2_O_3_ film and (**b**) YBCO film.

To verify whether YBCO grows epitaxially with texture on the Ni-W substrate and Y_2_O_3_ buffer film, Phi scan measurements were performed on the film surface. Y_2_O_3_ (440) and YBCO (103) reflections were chosen for in-plane Phi scan characterization. The in-plane texture of the Y_2_O_3_ and YBCO films is shown in Figure 9. Four-fold-symmetric diffraction peaks separated by 90° are observed without extra peaks. The FWHM values for Y_2_O_3_ and YBCO are 1.86°, 1.69°, 1.86° and 1.57° for the Y_2_O_3_ film and 1.98°, 1.78°, 2.03°, and 2.14° for the YBCO film, respectively, demonstrating that both Y_2_O_3_ and YBCO films achieve favorable in-plane epitaxial growth on the Ni-W substrate. The FWHM values of Y_2_O_3_ are lower than those of YBCO, revealing that Y_2_O_3_ possesses superior in-plane texture. It provides a high-quality textured template to guarantee the oriented growth of YBCO. Meanwhile, the FWHM values of YBCO are still below 2.5°, indicating that it inherits the in-plane texture from the Y_2_O_3_ barrier film, which lays a favorable crystallographic foundation for achieving a high critical current density (Jc).

**Figure 10:**
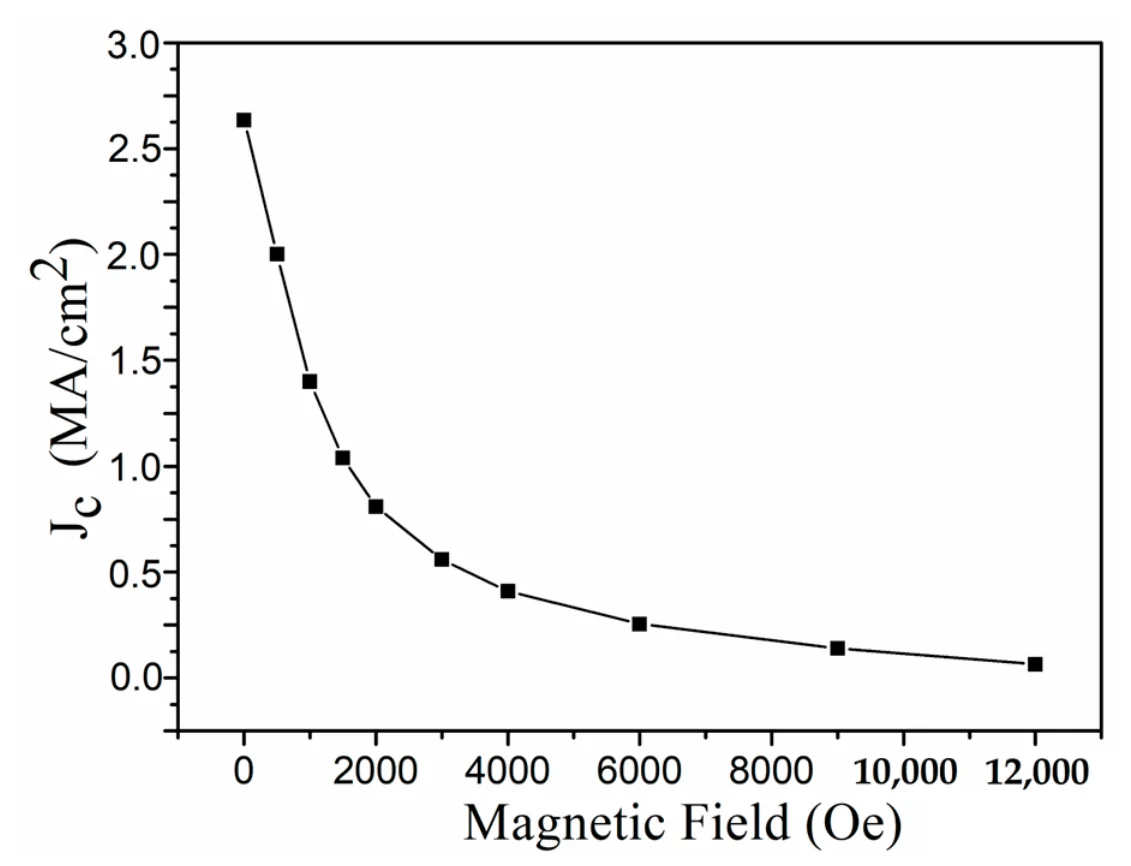
Jc curve of YBCO film under the different magnetic field.

Jc measurements were performed on the YBCO film with a well-developed oriented texture, and the field-dependent critical current density Jc at 77 K are shown in Figure 10. Under the self-field condition, the sample achieves a high Jc of approximately 2.65 MA/cm^2^. As the applied magnetic field increases from 0 Oe to 12,000 Oe, Jc decreases monotonically. A rapid degradation of Jc is observed in the low-field region of 0–2000 Oe, while the decay becomes moderate at higher magnetic fields. A finite Jc can still be maintained even at 12,000 Oe. Such behavior is typical of high-temperature superconductors, where flux creep leads to the suppression of current-carrying capability under a magnetic field. The high self-field Jc originates from the good biaxial texture of the YBCO film. Based on the YBCO film thickness of 0.68 μm and the tape width of 10 mm, the critical current Ic at 77 K was calculated to be 180 A/cm.

**Figure 11:**
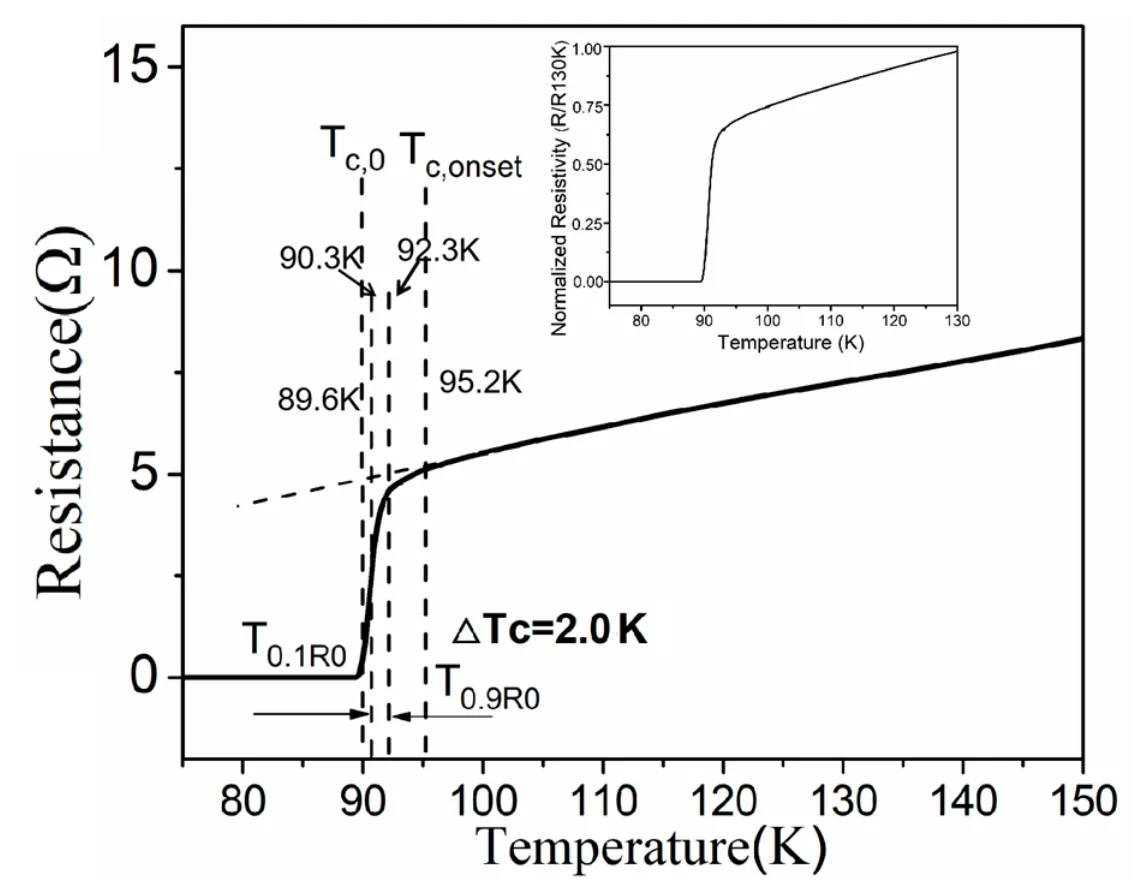
The resistance–temperature (R-T) curve and normalized resistivity inset of the YBCO film.

Figure 11 shows the normalized resistivity and resistance–temperature (R-T) and normalized resistivity curve of the YBCO-coated conductor measured by the four-probe method. At high temperatures, the resistance decreases approximately linearly as the temperature decreases. Upon further cooling, the resistance drops sharply and the superconducting transition occurs. The critical temperature is 95.2 K, and the YBCO superconducting transition width ΔTc, defined as the temperature difference between T_0.9R_ and T_0.1R_ (which correspond to 90% and 10% of the normal-state resistance, respectively), is about 2.0 K. The resistance finally falls to zero at the transition endpoint, indicating the fully superconducting state. The narrow transition width reveals high phase purity and good compositional homogeneity of the YBCO film.

In future work, we will systematically investigate the mechanical flexibility and strain tolerance of YBCO tapes with a single-layer Y_2_O_3_ buffer. Unlike conventional multilayer buffer architectures, this simplified structure reduces heterogeneous interfaces in coated conductors, which may relieve interfacial stress concentration and improve the mechanical integrity of superconducting films upon bending. Nevertheless, the bending fatigue, critical strain and structural stability under cyclic bending require quantitative characterization. Further mechanical tests will reveal the pros and cons of photochemically prepared single-layer Y_2_O_3_ buffers for flexible high-temperature superconductors and support the practical application of low-cost YBCO tapes.

## 4. Conclusions

The Y_2_O_3_ buffer film and YBCO superconducting film were fabricated on Ni-W substrates by combining photochemical and sol–gel methods. The following conclusions are drawn based on the FT-IR analysis of gel films as well as the structural and property characterizations of the heat-treated coatings.

1. UV photochemical treatment decomposes organic groups, removes residual carbon in the Y_2_O_3_ precursor films, promotes the formation of Y-O bonds, and mitigates defects induced by organic volatilization.
2. The UV-pretreated Y_2_O_3_ film has fine and uniform grains with greatly reduced surface roughness. A dense Y_2_O_3_ buffer film, free of pores or cracks and with good biaxial in-plane texture, is obtained after annealing at 900 °C.
3. The photochemically synthesized single-layer Y_2_O_3_ replicates the texture of Ni-W substrate and acts as a high-quality template for in-plane epitaxial growth of YBCO. YBCO inherits the underlying texture, with all FWHM values below 2.5°.
4. YBCO-coated conductor on this Y_2_O_3_ buffer film exhibits moderate superconducting transition width and a self-field Jc of 2.65 MA/cm^2^ at 77 K, with typical flux-pinning performance under magnetic field. This vacuum-free technique simplifies buffer-layer architecture and is promising for low-cost fabrication of YBCO-coated conductors.

## Figures and Tables

**Table 1:** The surface roughness of the Y_2_O_3_ buffer film.

Sample (50 μm × 50 μm)	RMS (nm)
No-UV-irradiated	19.8
UV-irradiated	5.46

## Data Availability

The original contributions presented in this study are included in the article. Further inquiries can be directed to the corresponding author.
